# Wear Analysis of Forging Tools Used in an Industrial Production Process—Hot Forging in Closed Dies of the “Head-Disk” of an Engine Valve Forging

**DOI:** 10.3390/ma14227063

**Published:** 2021-11-21

**Authors:** Marek Hawryluk, Jacek Ziemba, Marta Janik, Piotr Górski, Łukasz Dudkiewicz, Kornelia Głód, Jakub Krawczyk

**Affiliations:** 1 Department of Metal Forming, Welding and Metrology, Wroclaw University of Science and Technology, 50-370 Wroclaw, Poland; jacek.ziemba@pwr.edu.pl (J.Z.); piotr.gorski@pwr.edu.pl (P.G.); kornelia.glod@pwr.edu.pl (K.G.); jakub.krawczyk@pwr.edu.pl (J.K.); 2 Valves Department, Mahle Poland, Mahle 6, 63-700 Krotoszyn, Poland; marta.janik@mahle.com; 3 Schraner Polska, 99-100 Łeczyca, Poland; ldudkiewicz@schraner.pl

**Keywords:** valve forging, durability of forging punch, wear of calotte area, numerical modelling

## Abstract

The article performs an analysis of the durability of punches applied in the process of producing a valve forging from chromium-nickel steel. A forging of this type is made in two operations: coextrusion of a long shank, followed by finishing forging in closed dies of the valve head. The product obtained in this way (after other additional finishing procedures) constitutes the key element of the combustion engine (resistant to high pressures and temperatures) in motor trucks. Unfortunately, a significant problem in this production process is a relatively low durability of the forging tools, especially the punch used in the second forging operation. The key element at this stage, deciding about the punch’s further operation, is the area of the so-called “calotte”. The short-term life of the tools results from very hard performance conditions present during the forging process (periodical high mechanical and thermal loads, long path of friction). The latter cause intensive abrasive wear as well as high adhesion of the forging material to the tool surface. Based on the performed studies, including the following: technology analysis, numerical modelling, macro analyses combined with 3D scanning of tool sections as well as microstructural tests and hardness measurements, it was established that it is crucial to properly select the process parameters (charge and tool temperature, tribological conditions), as even slight changes introduced into them significantly affect the operation time of the forging tools. Mastering and proper implementation of the analyzed forging technology requires numerous further studies and tests, which will enable its perfection and thus increase the durability of the tools as well as the quality of the produced items.

## 1. Introduction

The exhaust valves of combustion engines in motor trucks usually operate at the temperatures of 700–850 °C, whereas, in the recent years, in order to increase the efficiency and capacity of the engines, the working condition temperatures have continuously been raised [1]. During the operation of such an engine, high pressures cause the exhaust valve to undergo cyclic thermal and mechanical loads [2,3]. Engine valves are usually produced by the method of hot plastic treatment from high-nickel austenitic steels, chromium-nickel steels or nickel superalloy of the Nimonic type (about 70–80% Ni content) [4]. These materials characterize in high corrosion resistance in combustion gases, high hardness as well as high abrasion resistance at high temperatures and high-temperature creep resistance [4].

**Figure 1:**
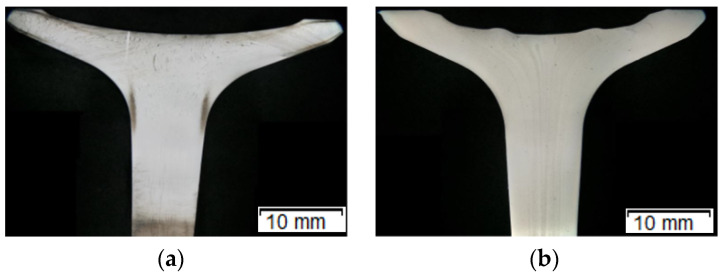
Photo with a macro-analysis of the fibre orientation in valves produced by way of: (**a**) upsetting and forging, (**b**) hot extrusion and forging [12].

At present, the valve production processes include two technologies based on hot plastic treatment [5,6]. One of them, commonly implemented, is a one-operation technology consisting in local heating of the charge material made of valve steel in the form of a small diameter bar, followed by local upsetting in order to obtain the proper shape of the so-called disk [7]. The other, less frequently applied, technology consists of two operations: hot coextrusion of a long shank tipped with a pre-formed element called “pear”, and next finishing forging of the valve head [8,9]. The first is well-mastered, yet relatively less cost-effective due to the cost of the charge material, as small diameter bars obtained by the method of drawing or extruding from wire rods are much more expensive (in respect of a bigger diameter obtained in a hot rolling process). Moreover, items produced via the first technology characterize in lower mechanical properties. The biggest effects of forgings obtained with the first technology are structural changes in the area of local heating (heat effect zone, large temperature gradients) during upsetting, causing cracking on the profile, as well as overheating and lapping of the forging disk (Figure 1a). In the case of the application of the second technology (forging with pre-extrusion, Figure 1b), the obtained products characterize in a homogeneous structure, as well as better mechanical properties (higher hardness) and surface quality [10,11].

The second technology is unfortunately randomly used, as it is a process of forging in closed dies, which is difficult to master because it requires the proper (often restrictive) selection of the key parameters: the appropriate charge mass, with the accuracy of up to 1%, maintaining a constant and repeatable temperature and lubrication conditions, as well as designing the proper shapes of dies and punches for a good filling of the impressions. In the case of the application of the second technology described above, during forging, very low pressures occur. This significantly affects the durability of the forging tools (in extreme cases, no more than 100 good forgings are made) and leads to the production of defective items [13]. It should be noted that the life of tools made of hot operation steel is the main problem in the case of shaping forgings from valve (austenitic) steels [14,15], as the mean durability of tools made of tool steels equals about 1000 austenitic steel forgings, about 5000 alloy steel forgings and even over 5000 low-carbon steel forgings [11,12]. One of the crucial problems during the production of austenitic steel forgings is increased adhesion of the charge material—chromium-nickel steel with the nickel content of 24–35% and chromium content of 14–20%—to the tool substrate (in the case of the first operation—extrusion—to the die, and the second operation—to the punch) made of tool steel [1]. This results from the difficulty in shaping steel with a high content of nickel and a poor selection of the process parameters [16,17]. As a result of high pressures at high temperatures as well as a long path of friction, in the case of the punch used in the second forging operation, we observe wear [18] (mainly abrasive wear as well as material tempering causing plastic deformation) of the so-called “calotte” on the head surface in the tool axis. This is the main cause of removing the punches from further operation (after they reach the critical dimensions of depth and calotte radius). Another cause of the tool’s removal are radial grooves running from the calotte outward (in the direction of the punch circumference). That is why, in the process, it is important to properly select the main technological parameters, e.g., tribological conditions [19] (proper charge and tool temperatures and cooling and lubrication agents), as well as the optimal shape of the forming tools. This should lower the forces during the shaping process as well as minimize the residual strains [20,21,22].

At present, for the analysis and optimization of industrial forging processes, a series of advanced engineering and IT (information technology) tools are used, e.g., a numerical method based on FEM [23,24] (Finite Elements Method), often combined with microstructural tests and hardness measurements, as well as thermovisual studies and a dimensional analysis with the use of laser scanners [25,26]. It seems, however, that the most information can be obtained from numerical modelling (with high experience and knowledge of the technologists), as computational packages of this type make it possible to determine many difficult to experimentally establish physical quantities as well as other technological parameters [22,27,28]. The combination of the mentioned research methods enables a complex analysis, based on which it is possible to solve many scientific issues and technological problems [29,30,31,32].

## 2. Materials and Methods

**Figure 2:**
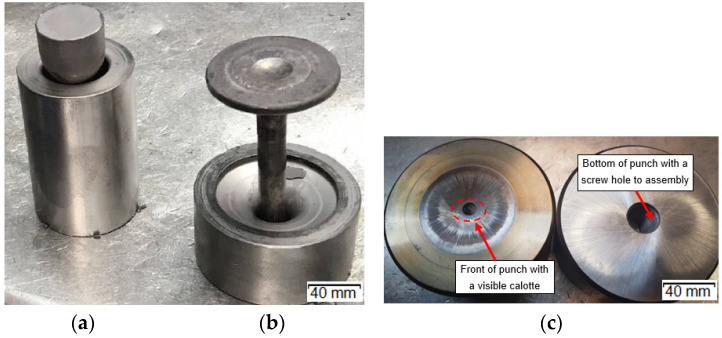
View of the forging and the die: (**a**) after the first operation, (**b**) after the second operation, (**c**) a worn forging punch after 3000 forgings; left—The working part with clear traces of abrasive wear on the surface and in the calotte area, right—The fastening part.

A durability analysis was carried out of the forging punches used in the hot production process of a valve forging from chromium-nickel steel (According to DIN; NCF3015 Table 1). A forging of this type is used as the key element of the engine in motor trucks. It is required to exhibit high performance properties, which is connected mainly with ensuring the proper flow of the forging material during forming, lack of surface and internal defects as well as high quality and dimension-shape precision. The forging is produced in two stages (operations). From a cylinder-shaped charge (with a diameter of 25 mm) heated to 1040 °C, in the first operation, a so-called “pear” is forward extruded (Figure 2a). Next, in the second stage, forging of the valve head takes place (Figure 2b) in closed dies.

All the forging tools in the analyzed process are made of W360 steels (Table 1).

**Figure 3:**
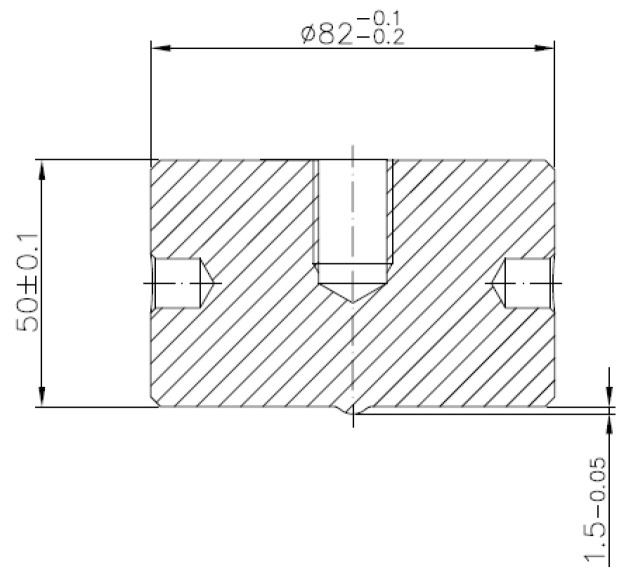
View of the schematic of the punch.

After thermal and thermo-mechanical treatment as well as finishing mechanical treatment, they obtain hardness of about 600 HV (about 53.5 HRC) for punches (Figure 2c) and over 1100 HV for dies (after nitriding of their surface in the depth of about 0.2 mm). The ready tools are heated to the working temperature of about 200–250°C before the start of the forging process. During the extrusion process, the tools (only dies in the first and the second operations, without punches) are lubricated and cooled with a lubricating-cooling agent based on graphite. A detailed analysis was performed of the forging punch (made of W360 steel, Figure 2c) used in the second operation—finishing forging—due to its relatively low durability, especially in the vicinity of the calotte (Figure 3), as a result of increased adhesion of the charge material to the tool substrate.

In order to perform a complex analysis, the following research methods were applied:

- Macroscopic studies combined with a measurement of the degree of wear/excess on the tool’s working surface through scanning by means of a measuring arm ROMER Absolute ARM 7520SI (Hexagon Manufacturing Intelligence, Cobham, UK) integrated with a RS3 scanner (Hexagon Manufacturing Intelligence, Cobham, UK), as well as a comparison of the scan geometry with the CAD models (Version number 1.5, Krotoszyn, Poland), and also reconstruction of the wear history of the key area, the “calotte”, based on reverse scanning; all punches were cleaned before analysis.

A representative section of the sample was then cut from the punches and the study continued:

- Observations of the destruction features on the working surface by means of a scanning electron microscope LEO 1430 coupled with an EDX detector (Zeiss, Jena, Germany);

Prior to the preparation of the metallographic section, photographs were taken on a stereoscopic microscope:

- Microstructural tests of the surface layer in the tool axis made by means of a stereoscopic microscope Keyence VHX-S600E (Osaka, Japan) and a light microscope Olympus BX51M (Tokyo, Japan); after the preparation of samples and microsections, the tools were etched in 4% nital (grinding and polishing on a grinder-polisher Struers 350 (Struers, Rødovre, Denmark); microhardness measurements in the cross section, in the function of the distance from the surface, made with the use of a hardness tester LECO LC100 (LECO Corporation, St. Joseph, MO, USA) in order to evaluate the degradation occurring on the tool surface caused by the operation of high temperatures during forging;
- Analysis of the chemical composition made by the method of glow discharge optical emission spectroscopy (LECO Corporation, St. Joseph, MO, USA) by means of an analyzer GDS 500A by LECO (LECO Corporation, St. Joseph, MO, USA);
- Numerical modelling was used to simulate the wear mechanisms of the punches by means of the Forge NxT 3.0 computational package (Transvalor, Biot, France).

## 3. Analysis of Results

**Figure 4:**
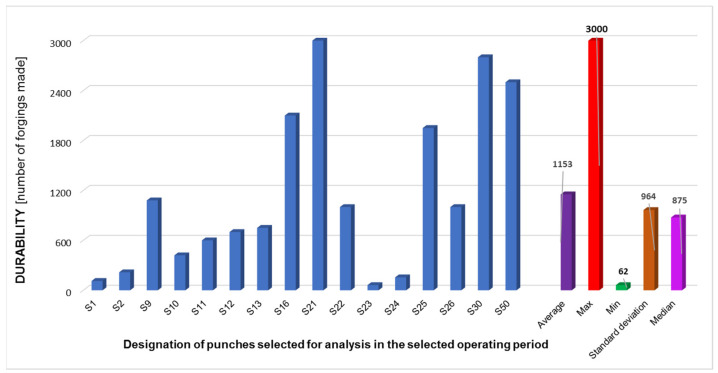
Comparison of the durability of selected punches (S1–S50) used in the second forging operation during the time of 10 months.

In the first place, an in-depth analysis of the production process was performed, especially in reference to the second forging operation and the wear of the punches used in this operation. The long-term analyses of the process demonstrated lack of stability of the performance time of these tools, the main cause of which were probably non-optimal tribological conditions. Long production cycles were observed for the punches used in the second operation, even about 3000 forgings, as well as short cycles, 200–700. The observations also included the total wear of the die in the first operation as a result of friction after 1–2 forgings. Figure 4 shows a compilation of durabilities for selected representative tools (forging punches) for the time period of 10 months. In the case of punches that performed a small number of cycles, the most common reason for this was the plastic deformation of the stamp’s calotte, noticed by the operators, which disqualified the tool from further production.

**Figure 5:**
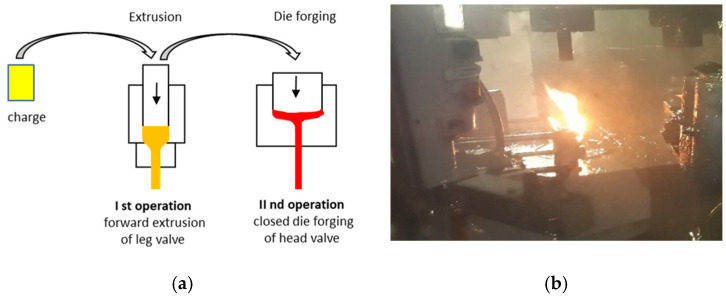
View of: (**a**) a diagram of the analyzed hot extrusion and forging technology, (**b**) a photograph of the press chamber in an industrial forging process with visible flames resulting from burning out of the oil in contact with the hot forging in the die.

Based on the conducted analysis, we can observe that the average durability of the punches in the selected time period equals about 1150 forgings, where we should note the high value of standard deviation. It was observed that, among all the tools (except for the punches which produced a dozen or so forgings, usually during the production initiation), we can distinguish between two groups, with low durability in the scope of 400–950 forgings and high durability in the scope of much over 2000 forgings. What is more, after the whole production cycle, large amounts of lubricant residue in the working cabin of the press were observed. These suggest improper settings of the lubrication-cooling system (Figure 5). And so, we can conclude about significant possibilities, if not a necessity, to optimize the analyzed technology [33].

The observations as well as analysis of the selected technology make it possible to confirm that it is difficult to master. The forging process has low stability and requires the selection of optimal technological parameters, as even their slight change can significantly affect the forging quality and tool durability.

### 3.1. Macroscopic Analysis

**Figure 6:**
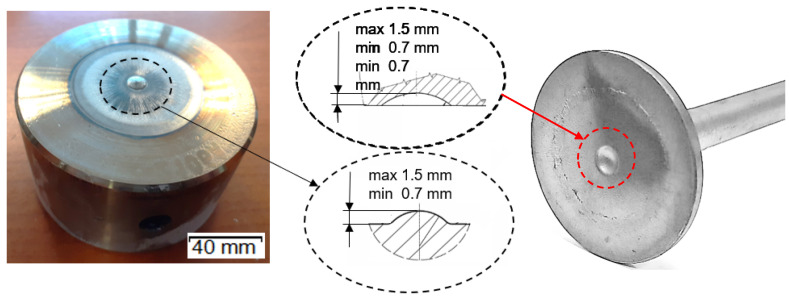
Pictures of a punch with the marked calotte—the key area determining a removal of the tool from further production (**left**) and view of the “head-disk” surface of the valve forging (**right**).

In the case of punches, the so-called “calotte” is crucial (Figure 6), because, when its shape or assumed dimension are changed, the tool is withdrawn from the process. In the case of a new stamp, the nominal value of the calotte’s height is 1.5 mm. During the realization of the process, the punch calotte is imprinted on the front surface of the forging disk, leaving a characteristic spherical cavity, which is controlled by the operator during the process. If the depth of the calotte’s imprint in the forging head after the second operation is less than 0.7 mm, the process is interrupted and the punch is replaced with a new one.

**Figure 7:**
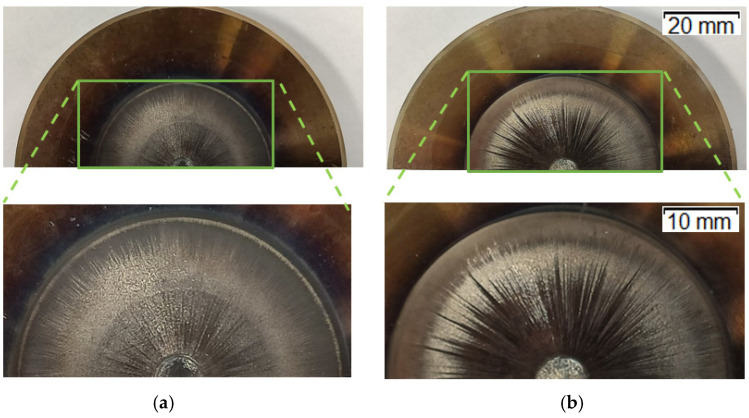
Macro-images of the punches with magnification of the working section: (**a**) a punch after producing 700 forgings, (**b**) a punch after producing 3000 forgings.

Figure 7 shows images of the two representative groups of punches (with magnification of the front working surface), which worked over 700 forgings (Figure 7a) and over 3000 forgings (Figure 7b).

While analyzing the images of the worn tools (Figure 7), we can notice that the punch after 700 forgings is slightly less worn. However, we can see numerous radial grooves running from the calotte outwards, as well as plastic deformation of the calotte combined with abrasive wear. In turn, in the case of the punch that worked over 2500–3000 forgings, the grooves are much deeper and longer, as they reach the end of the working section. The calotte is also strongly worn, and, additionally, we can observe that the working section is darker (burned). This can also prove the occurrence of oxidation of the working surface as a result of multiple contacts with the hot forging. Despite the fact that the punch after 700 forgings was less worn, it was removed from production due to unacceptable geometrical changes caused by the wear—the control dimension of the calotte’s height exceeded the limit value acceptable for the process.

### 3.2. Dimensional Analysis of Calotte Wear with the Use of 3D Scanning and the Reverse Method

For the investigations, the Polyworks software was used together with a measuring arm ROMER Absolute ARM 7520si integrated with an RS3 scanner. The ROMER Absolute ARM 7520si gives the possibility to collect up to 460,000 points/s for 4600 points on the line with the linear frequency of 100 Hz. The accuracy of the Absolute ARM 7520si according to the norm B89.4.22 equals 0.053 mm, according to ISO 1:2016.

**Figure 8:**
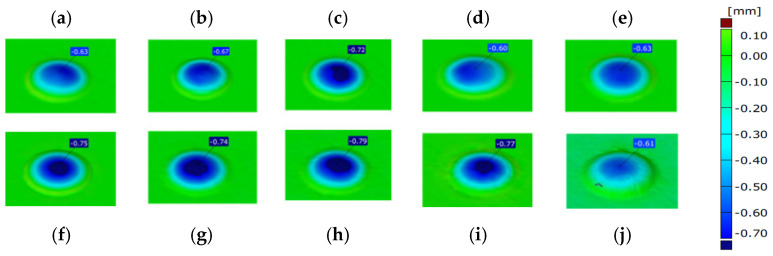
Results of 3D scanning of the punch calotte after the increasing number of produced forgings: (**a**) 62, (**b**) 420, (**c**) 600, (**d**) 620, (**e**) 700, (**f**) 1320, (**g**) 2100, (**h**) 2320, (**i**) 2660, (**j**) 3000 items.

**Figure 9:**
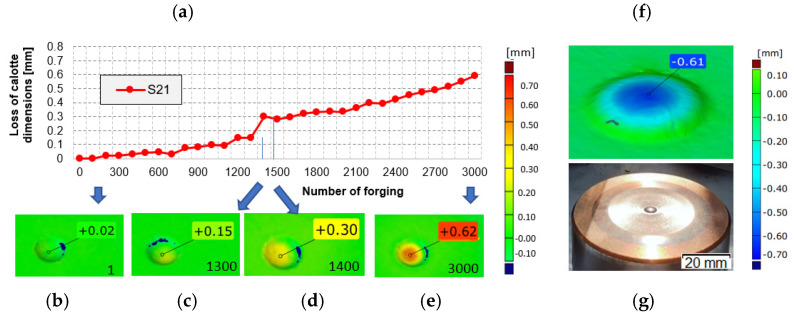
(**a**) Results of reverse scanning of the changes in the punch calotte’s imprint depth in the forgings: (**b**) 1st forging, (**c**) 1300th forging, (**d**) 1400th forging, (**e**) 3000th forging, (**f**) a calotte scan at the end of operation, (**g**) image of the punch.

The performed analysis of the punch’s 3D scanning results (Figure 8), especially the height measurements of the calotte (which is crucial for the realized process), makes it possible to establish and confirm that, in the analyzed case, the most common cause of removing the tool from production is a lowered height of the calotte by a value over 0.7 mm. In most of the analyzed cases, the limit dimension was exceeded (dark blue area), which resulted in removal of the tool and interruption of the forging process. The scanning results of a tool removed at the end of its operation do not, however, enable an evaluation of the course of the calotte’s wear during the forging process. For this reason, it was decided to collect forgings from the forging process in a cyclic distance of 100 items. By the application of the reverse scanning method (developed by the authors), it is possible to determine the change in the calotte’s height based on the measurements made by means of the 3D scanning method [34,35,36]. Figure 9 shows an exemplary diagram of the changes in the calotte’s height for the cyclically collected forgings from the punch that produced 3000 forgings.

**Figure 10:**
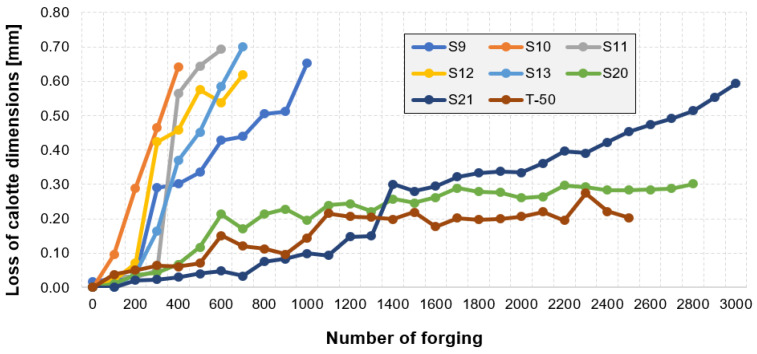
Diagram of the calotte’s height loss by the reverse scanning method from cyclically collected forgings for selected punches.

An analysis of the diagram (Figure 9a) makes it possible to interpret the calotte’s height loss for a single tool in time. The jump in the wear between 1300 and 1400 forged parts is probably caused by the process being stopped due to the necessity of changing the die-tool in the first operation and then restarting the whole process. In order to elaborate a full analysis of the calotte’s wear in time for the other punches, it was decided to carry out a similar analysis by means of the 3D reverse scanning technique. Based on periodically collected forgings (every 100 items) for each tool, measurements of the calotte’s imprint depth in the forging were made, which is adequate to the real wear of the calotte on the punches (Figure 10).

While analyzing the presented diagram, we can notice (and this also confirms the previous analyses referring to the wear of the selected punches) that the results are arranged in two characteristic groups. In the first group of punches, a relatively fast loss of calotte’s height takes place, which is the cause of their premature removal from the production process. The punches make it possible to obtain from 200 up to 900 forgings fulfilling the process requirements. The remaining tools belong to the second group characterizing in higher hardness. The material loss in the analyzed cases is almost linear and very slow. In the case of S21, we can notice that, after the production of 3000 forgings, the height loss was at the level of 0.5 mm (if the tool had been continuously used, it would have probably produced about 4200 forgings). Similarly, in the case of punch S30, after 2800 forgings were made, the height was reduced to only 0.3 mm (also in this case, if the tool had operated further, it would have probably produced about 6500 forgings). In the case of T-50, the loss was at the level of 0.2 mm, after the production of 2500 forgings (analogically, in this case, if the tool had been used further, it would have enabled the production of about 9500 forgings). In the case of the analyzed group of tools, we find that they were prematurely removed from the production process. It may be that the cause of that were other damages connected with the calotte, e.g., too deep radial grooves or yet other technological reasons. Also, it should be noted that the operators considered the average punch durability at the level of 1500 forgings, and for these tools, damage or wear could have been observed for the die in the first or second operation. In such a case, according to the assumed technology, the whole set of tools is replaced.

Nevertheless, on this basis, it is difficult to state why that group of punches worked over a shorter period of time (200–700 forgings) and as a result of the calotte’s wear, it was removed from production. In turn, a group of a few punches worked 2–3 times longer (over 2400 forgings), and additionally, the calotte was still within the scope of acceptable value of height control dimension. That is why, for further analyses related to microstructural investigations, one representative punch was selected for each of the two groups.

### 3.3. Analysis of Microstructure and Topography of Punch Working Surfaces

**Figure 11:**
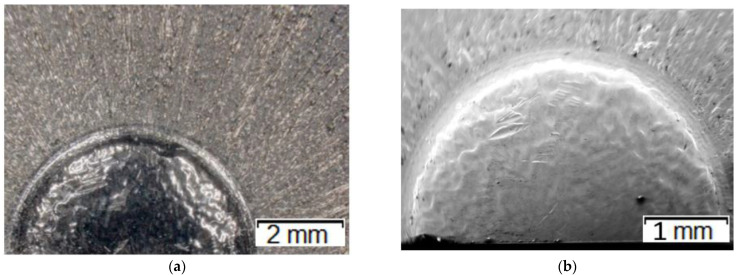

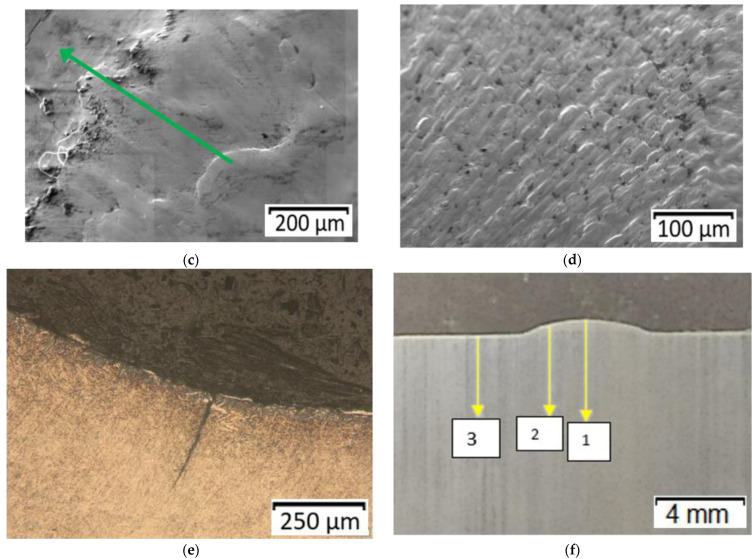
Microstructural analysis of the punch which produced 700 forgings: (**a**) macro-image of the calotte, (**b**) SEM analysis of the calotte (low magnification), (**c**) SEM image of the calotte—visible plastic deformations (green arrow shows the direction of plastic deformation), (**d**) SEM of the front surface—thermo-mechanical fatigue, (**e**) a micro-crack at the base of the calotte, (**f**) vectors of the hardness measurements.

Figure 11 shows the results of microstructural analyses of the calotte as well as the adjoining area together with the marked zones of microhardness measurements for the punch which worked over 700 forgings. Additionally, Figure 11d shows thermo-mechanical fatigue (scale-shaped surface).

The performed analysis made it possible to observe traces of wear on the calotte’s surface, as well as scratches and grooves. That proved progressing wear, including not only the typical abrasive wear but also thermo-mechanical fatigue, plastic deformation, as well as fatigue cracking at the calotte’s base (Figure 11e). Based on the preliminary microstructural tests, it was established that, on the working surface of the worn punch, especially in the selected characteristic areas, we can notice clear traces of adhesion, i.e., sticking of the forging material, as well as cracking of the tool material and detaching of larger fragments. This, in turn, results in traces of abrasive and thermo-mechanical wear. The areas of microhardness measurements are shown in the article in Figure 11f. The measurements were made for two representative punches: which worked over 700 forgings (Figure 7a) and over 3000 forgings (Figure 7b).

**Figure 12:**
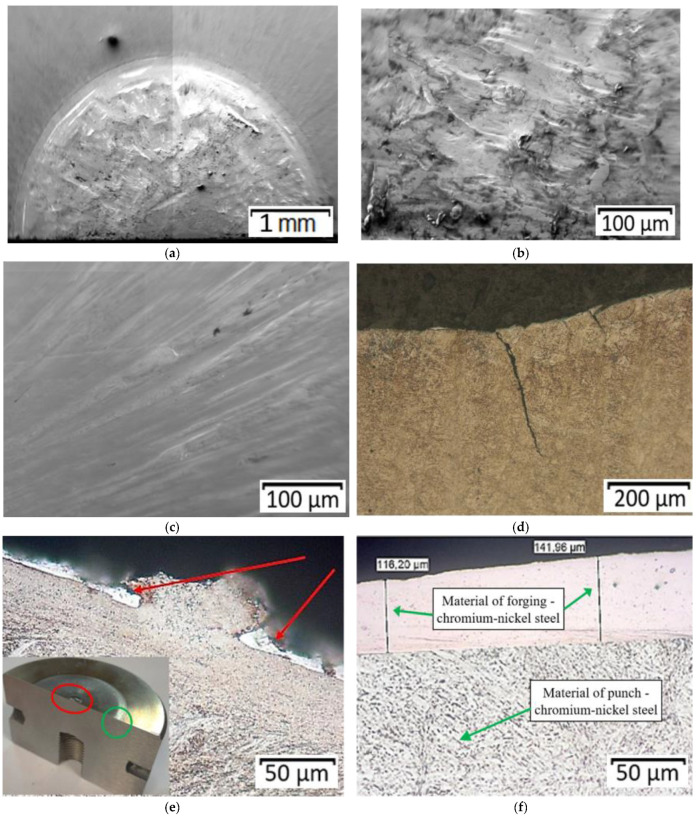
Images of: (**a**) area of the punch’s calotte with visible traces of plastic deformation, (**b**) SEM from the punch calotte area (marked with a red circle in Figure 11e) with a zone of grooves and stickings, (**c**) SEM from the selected area (green)—visible stickings of the forging material (**d**) area of calotte base—mechanical crack, (**e**) calotte—crack of the surface layer, (**f**) area of punch—sticking the forging material to the surface of the tool.

The punch that worked over 3000 forgings (Figure 12) underwent tempering in the calotte, while, on the front surface, no tempering was detected.

Similar to the case of the punch that worked over 700 forgings, we can observe fatigue cracks near the calotte and numerous traces of sticking of the forging material on the working surfaces (Figure 12e,f) as well as a residue of the oxidation process.

### 3.4. Microhardness Measurements

**Figure 13:**
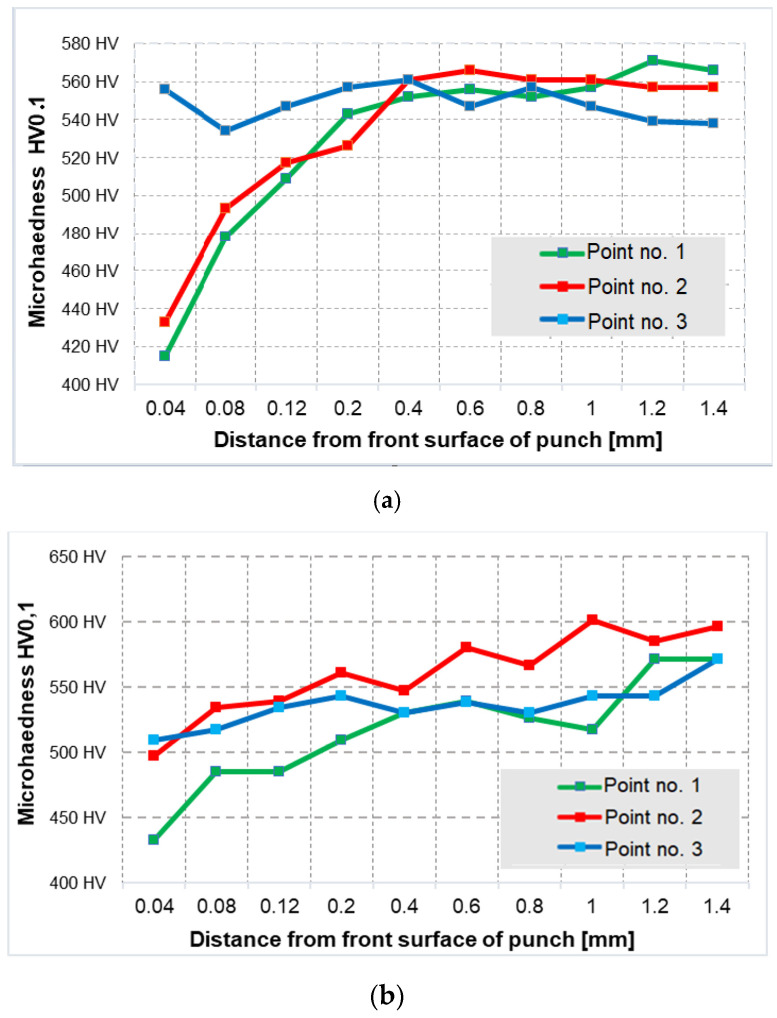
Course of hardness for the selected two representative punch groups (**a**) punch after 700 items, (**b**) punch after 3000 items.

For a fuller analysis of the topographic and structural changes, microhardness measurements were made for both representative punches (the measurement areas marked in Figure 11f). The obtained results in the 3 analyzed areas for both punches have been presented in Figure 13.

The hardness measurement results show that the punch that worked over 3000 forgings underwent tempering in the calotte, whereas the punch that produced 700 forgings was tempered both in the calotte and on the front surface (a visible drop of hardness on the front surface proving tempering of the tool during its operation). On this basis, it is difficult to clearly state why both representative tools (the same geometry, material, hardness and process) behaved differently during the forging process and why they characterized in decidedly different operation times (periods). However, the fact remains that the production technology has not been fully mastered. Moreover, based on numerous analyses of the industrial process, its instability was established, probably resulting from the selection and assumption of non-optimal technological parameters. That is why the following stage of research was numerical modeling of the forging process (especially the second operation) for different variants of charge and tool temperature as well as friction.

### 3.5. Numerical Modelling

In order to more thoroughly determine the potentially crucial factors affecting the forging process, a multi-variant numerical analysis was conducted for the presently realized technology (for the second operation). The conditions were assumed in accordance with those of the industrial process, and next numerical modelling was carried out for several other variants—slightly altered tribological conditions in respect of the nominal technology. The most probable process variants that might occur under industrial conditions were selected, connected with the difficulties in controlling the process parameters or poor implementation of the difficult technology. Such an approach is justified because, as a result of a virtual experiment (FEM), we can, in a relatively short time, determine or describe such quantities and parameters which are impossible to determine/define in another way. What is more, conducting studies and tests under industrial conditions for different variants could cause significant difficulties as well as shutdowns in the current production, and thus also economical losses.

## 4. Assumptions and Boundary-Initial Conditions for Numerical Modelling

In order to determine the limit values of the effect of temperature and tribological conditions on the course of the forging process, numerical simulations in the FORGE 3.0NxT program by the finite element method were carried out. The following boundary conditions were assumed for the simulation, based on the nominal process realized under industrial conditions:

- Nominal temperatures: charge—1040 °C, tools—200 °C, environment—30 °C;
- Press Metal Press700 E (characteristics selected from the program database);
- Friction model according to Tresca; assumed coefficient of friction = 0.4.
- Thermal conductivity coefficient in contact—10 kW/m^2^·°K (denoted as medium in the program) and thermal conductivity coefficient with the environment—15 W/m^2^·°K (determined experimentally);
- The time of transport from the first to the second operation was assumed together with the time from the moment of forging—2 s.

**Figure 14:**
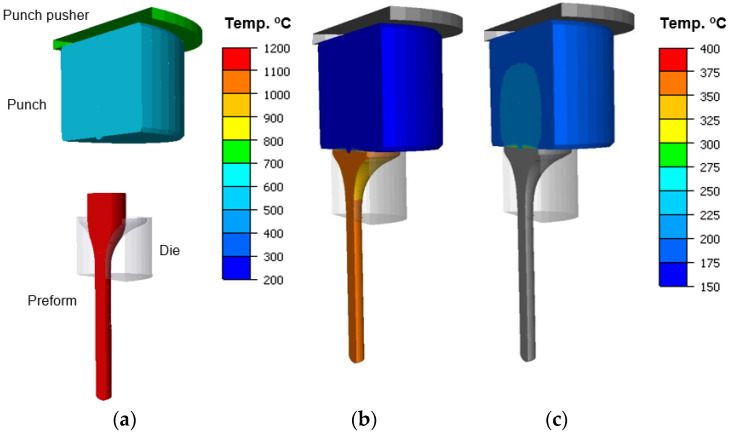
The view of: (**a**) the tool arrangement for simulations after the first operation, (**b**) after the second operation with the temperature scale for a forging, (**c**) after the second operation with the temperature scale for a punch; the results for the simulation were implemented from the first operation for the nominal process.

In the simulation of forging a valve in the second operation from a preform in the shape of a “pear”, an axisymmetric model was assumed with deformable tools-punches, whereas the dies and the pusher were assumed as non-deformable. Only the of extrusion was simulated, while, for each simulation variant, all the results were collected from the first forging operation. As the forging material, steel 1.4981 (X8CrNiMoNb16-16) was applied, which, according to software base, demonstrates similar mechanical properties to the charge material used in the real process. As the punch material, steel WCL was applied (similarly to the forging process). Figure 14 shows a general view of the model before and after the simulation of II forging operation.

During the multi-variant numerical simulations, five different variants were simulated:

I—Process assumed according to the technology, i.e., charge temperature 1040 °C, tool temperature 200 °C, friction according to Tresca factor: m = 0.2;

II—Charge temperature lowered to 940 °C, the remaining quantities assumed as for the process according to the technology (I variant);

III—Tool temperature increased to 300 °C, the remaining values as in I variant;

IV—Process assumed according to the technology, yet with friction increased to m = 0.6;

V—Charge temperature increased to 1140 °C and friction increased to m = 0.6.

A detailed analysis was performed of the distributions of temperature fields, forging forces and total wear for the selected five variants, by way of referring the results to the presently realized technology—variant II.

**Figure 15:**
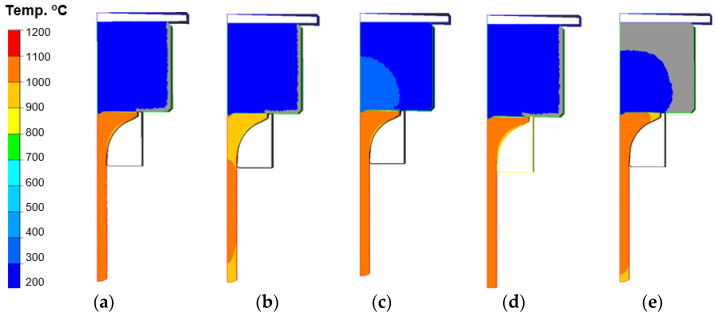
Temperature distributions for the selected variants at the final stage of extrusion: (**a**) I—Nominal variant, (**b**) II—Variant, (**c**) III—Variant, (**d**) IV—Variant, (**e**) V—Variant.

Figure 15 shows the temperature distributions for the selected variants at the final stage of the process. Based on the presented results with the temperature distributions for the punch, we can observe that, in all the cases, except for the tool temperature increased to 300 °C, the external surfaces of the punches underwent cooling below 200 °C—to about 180 °C. In turn, for variant III, with an increased temperature of the punch, we can additionally observe that the higher initial temperature of the tool caused an increase in the temperature in the central section on the side of the contact with the forging to about 400 °C.

**Figure 16:**
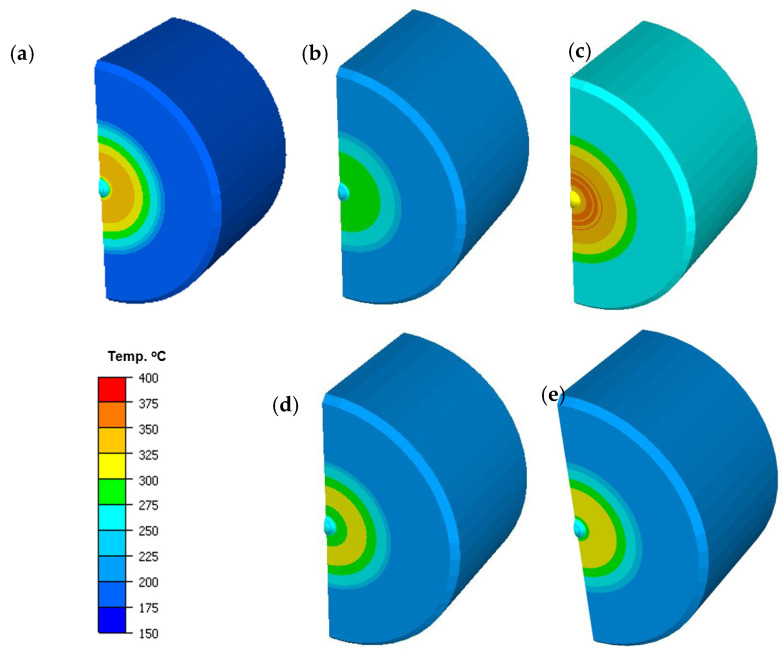
Temperature distributions on a punch’s section (for the half model from the axisymmetric system) for the selected process variants: (**a**) I nominal variant, (**b**) II, (**c**) III, (**d**) IV, (**e**) V.

In turn, in the case of temperature distributions in the forgings, after this operation, we can observe that, for all the variants, except for the charge temperature lowered to 940 °C, the mean temperature equals about 1100 °C. For variant V, both for the punch and the forging, we can notice a temperature drop of about 100 °C. A probable cause of this could have been increased friction, which resulted in a hindered flow of the forging material within the disk section and, in consequence, a longer contact with the punch, which received more heat from the hotter forging. Figure 16 shows the temperature distributions on a punch’s section on the side of the contact with the forging (disk section) for the particular variants (for the half model from the axisymmetric system) towards the end of the forging process, for a better analysis of the temperature changes in the contact. While analyzing the temperature distribution fields, we can state that, except for the punch for which the temperature was increased to 300 °C, no significant differences are visible. In turn, it should be noted that, during the analysis of the temperature field changes, some differences could be observed. One of them was the fact that, for the punch in the process of increased friction, a longer time of contact with increased temperature took place in the vicinity of the calotte. In the case of variant V, we can observe that the area from the calotte to the spot of intensive abrasive wear and adhesion has the highest temperature, about 250 °C, compared to the others, with the obvious exception of variant III (higher initial temperature of the punch). It should also be emphasized that the performed studies of the temperature distributions on the tools, including the punch, showed that the temperature at the distance of 10 mm from the working surface of the calotte during the process is higher than that assumed in the technology, and it equals 280 °C.

**Figure 17:**
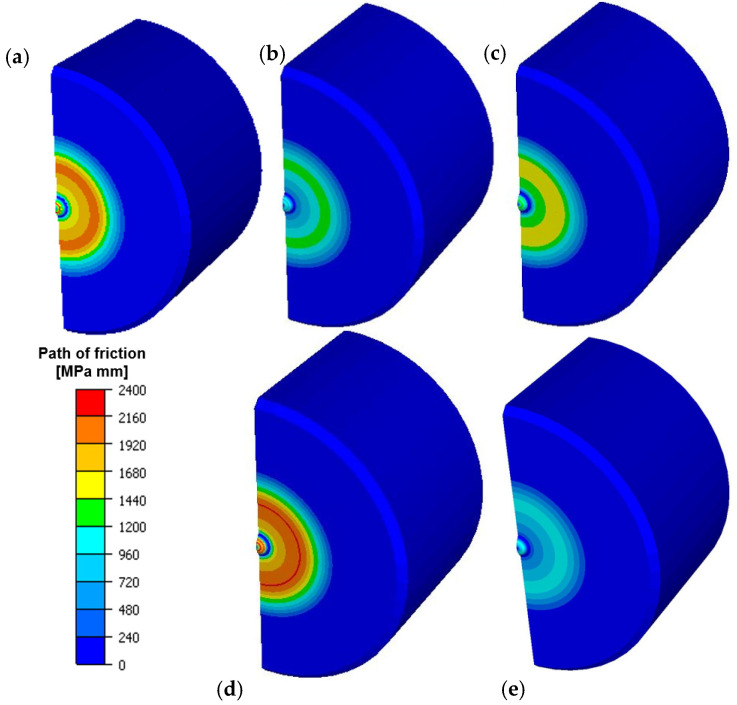
Distributions of the total abrasive wear for the selected simulation variants: (**a**) I, (**b**) II, (**c**) III, (**d**) IV, (**e**) V.

This confirms the fact that the analyzed technology still requires a lot of analyses and tests so that additional, confirmed, information can be collected, based on which it will be possible to take proper remedial measures. Figure 17 shows results of numerical simulation with distribution of abrasive wear.

On the basis of the presented results, we can notice that the highest total wear took place for the variant with the temperature on the punch increased to 300 °C. Such a situation can cause lowering of the yield point for the forging material receiving more heat in the contact from the punch. This probably favours a better flow of the forging material along the tool and causes its increased wear. For the remaining variants, the distributions are basically similar; only for the tool variants with increased friction, the abrasive wear is slightly lower, which may be caused by a hindered flow of the material along the punch and thus its lower wear. The highest wear values are observed on the calotte and at a certain distance from the punch axis in a radial direction (outside the material), in the area where increased abrasive wear and adhesion were observed during the macro tests.

**Figure 18:**
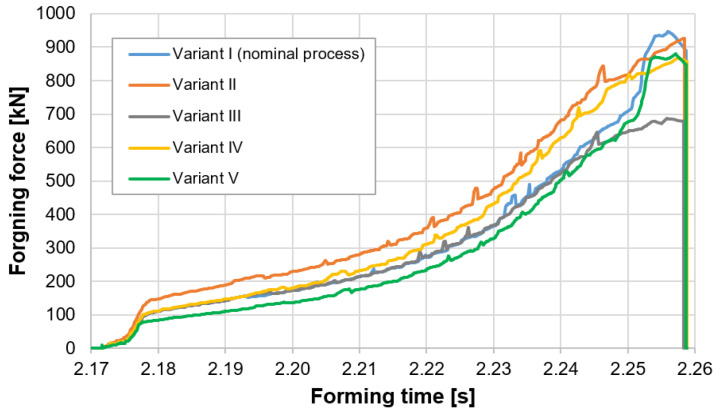
Courses of the extrusion forces in the function of deformation time.

Figure 18 shows the courses of the forging forces in the function of the deformation time.

Based on the presented courses, we can see that the highest force values were obtained for the variants: I—Nominal process and II—Forging temperature lowered to 940 °C. In turn, the lowest values were achieved for variant III—Tool temperature increased to 300 °C and variant IV—Friction increased to 0.6.

For variant IV, the force value oscillates at the level of 900 kN. In the case of variants III and IV, the lowering of the maximal force in respect of the nominal process (variant I), as well as the lowering of the initial temperature of the tools in variant II, can be explained by a slightly different way of deformation of the forging material in the second operation as a result of changed tribological conditions. It is worth considering the fact that, while the increased friction in respect of the nominal process can cause such a course, increasing the tool temperature to 300 °C can cause increased heating of the forging material, and thus lower the yield point. However, it is a difference of about 25% in respect of the nominal process. A similar situation can be noticed for variant III, where a higher initial temperature of the punch (300 °C) causes increased friction.

**Figure 19:**
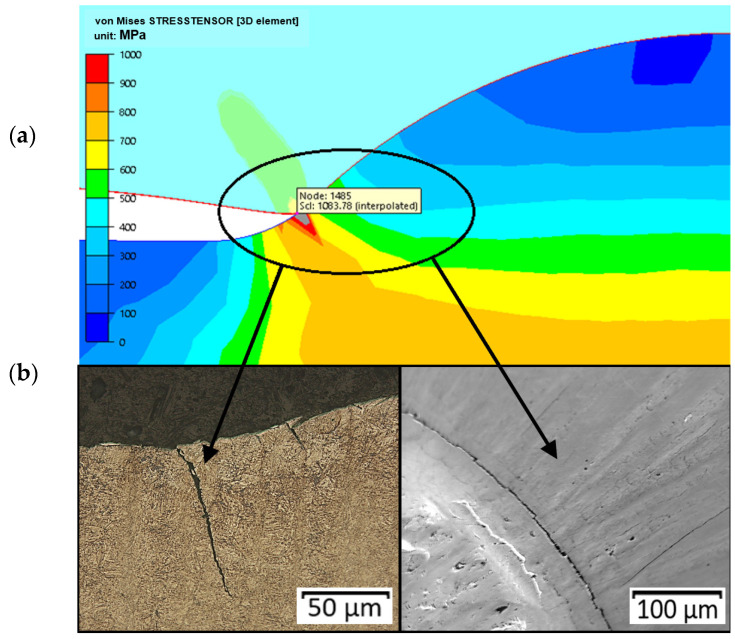
A crack localized in the area of stress concentration on the circumference of the calotte: (**a**) results of numerical simulations (top—The forging’s disk, bottom—The punch’s calotte), (**b**) SEM and a microstructure image.

During the numerical modelling referring to an analysis of the reduced stresses, a concentration of stresses was observed, especially in the initial phase of the process (in the second operation). This stress concentration can cause the formation of cracks observed in this area, shown in Figure 19. We can notice that the crack runs according to the direction of the operation of the highest reduced stresses.

**Figure 20:**
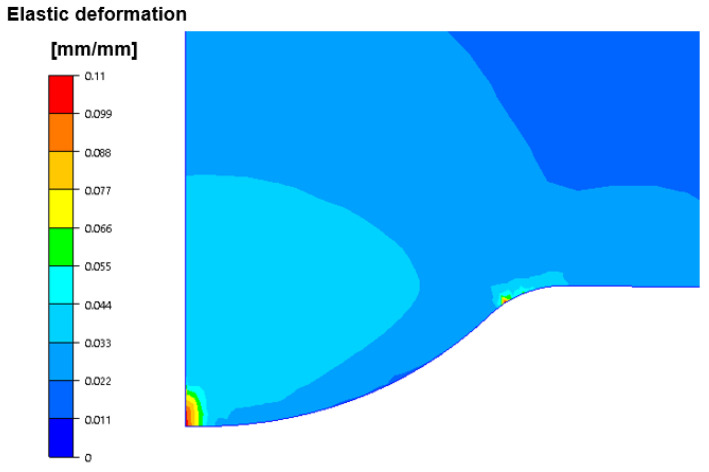
Distribution of plastic deformations on the punch after second forging operation.

We can notice that the crack runs according to the direction of the operation of the highest reduced stresses. The modelling also included an analysis of the possibility of the occurrence of plastic deformation, which can appear as a result of high pressures as well as a long contact of the tool with the formed forging material. Figure 20 presents the calculated distributions of plastic deformations in the area of the calotte on the punch from II operation of the valve forming.

As we can notice, based on the simulation results, the plastic deformations are localized in the center of the calotte and at its base, which is in the area where a high concentration of stresses takes place at the beginning of II operation as well as in its last phase. For this reason, the calotte can change its geometry at the top (it can plastically deform) or undergo abrasive wear. In turn, at the base of the calotte, fatigue cracks may occur, which was also confirmed by the microstructural analyses.

The performed multi-variant analysis of the industrial process (2 operations) of producing a valve forging with the use of numerical modelling provided a lot of valuable information, which would be difficult to obtain in an analysis of the industrial process. This referred to the distribution of temperature fields and pressures as well as the parameters describing the wear for changing conditions, and also the courses of the forging forces. The performed computer simulations confirmed the results of the macroscopic observations pointing to the weak points of the tool wear. On this basis, we can also state that even small changes in the key tribological parameters or lack of their constant value in the process, such as the charge and tool temperature as well as lubrication, can cause premature wear of the tools. Unfortunately, due to the high production costs, it is difficult to carry out long-term tests under industrial conditions for selected, most possible variants, other than the assumed nominal variant, i.e., adopted as the correct technology for manufacturing the valve forgings.

## 5. Conclusions

The article presents the results of investigations referring to the wear of punches used in the second operation of producing a valve forging from chromium-nickel steel. The conducted analysis demonstrated that:

- The realized technology is very complicated under the industrial conditions because even slight changes in the optimal key technological process parameters can cause premature wear of the tools,
- The conducted FEM analyses made it possible to establish that a change in the three selected parameters: the charge temperature 1040 °C in the change scope of ±100 °C, the tool temperature 200 °C in the change scope of ±100 °C and the friction m = 0.2 ± 0.4, negatively affects the analyzed process,
- After considering the results of numerical modelling and introducing some corrections into the industrial process, it will be possible to improve or even optimize the present technology with the aim to increase tool durability,
- Using the current numerical methods combined with microstructure studies assisted by other techniques, it is possible to design or verify and improve the technology of production through die forging.

Also, more research is being conducted on the improvement of the demonstrated technology, which includes numerical modelling with the consecutive variants of tribological parameters, in order to select the most optimal parameters with the purpose to increase the durability of forging instrumentation. In turn, under industrial conditions, verification is being made of the thermal parameters connected with following the technology of tool and charge heating, as well as the manner of lubrication, including a change of the lubricating agent. Future plans involve the application of hybrid layers as well as implementing changes in the parameters of the tool surface layer or changes of the tool material. In addition, due to the observed high adhesion of the forging material to the tool, the authors plan to conduct detailed microstructural studies of the charge material after heating it to the forging temperature, in order to analyze the microstructural changes, including: complete dissolution of carbide precipitates in NCF 3015 steel.

## Figures and Tables

**Table 1:** The chemical composition of the input material (NCF3015) and tools (W360) [%].

Elements	C	Si	Mn	P	Ti	Ni	Mo	Al	Cr	Nb	Cu	V
NCF3015	0–0.08	0–0.50	0–0.50	0–0.015	2.30–2.90	30–33.50	0.4–1.00	1.6–2.20	13.5–15.5	0.40–0.90	max 0.5	-
W360	0.50	0.20	0.25	-	-	-	3.00	-	4.50	-	-	0.60

## Data Availability

Data sharing not applicable.
